# Ethyl 3-(9-chloro-10-oxo-9,10-di­hydro­anthracen-9-yl)-5-methyl­isoxazole-4-carboxyl­ate

**DOI:** 10.1107/S1600536814003080

**Published:** 2014-02-19

**Authors:** Nathan S. Duncan, Howard D. Beall, Alison K. Kearns, Chun Li, Nicholas R. Natale

**Affiliations:** a Department of Pharmaceutical & Biomedical Science, The University of Montana, 32 Campus Drive, Missoula, MT 59812, USA; b Department of Chemistry, Ithaca College, 953 Danby Road, Ithaca, NY, 14850, USA

## Abstract

The asymmetric unit of the title compound, C_21_H_16_ClNO_4_, contains two independent mol­ecules (*A* and *B*), each adopting a conformation wherein the isoxazole ring is roughly orthogonal to the anthrone ring. The dihedral angle between the mean plane of the isoxazole (all atoms) and the mean plane of the anthrone (all atoms) is 88.48 (3)° in one mol­ecule and 89.92 (4)° in the other. The ester is almost coplanar with the isoxazole ring, with mean-plane dihedral angles of 2.48 (15) and 8.62 (5)°. In both mol­ecules, the distance between the ester carbonyl O atom and the anthrone ketone C atom is about 3.3 Å. The anthrone ring is virtually planar (r.m.s. deviations of 0.070 and 0.065 Å) and adopts a shallow boat conformation in each mol­ecule, as evidenced by the sum of the six intra-*B*-ring torsion angles [41.43 (15) and 34.38 (15)° for molecules *A* and *B*, respectively]. The closest separation between the benzene moieties of anthrones *A* and *B* is 5.1162 (7) Å, with an angle of 57.98 (5)°, consistent with an edge-to-face π-stacking inter­action. In the crystal, weak C—H⋯O and C—H⋯N inter­actions link the mol­ecules, forming a three-dimensional network.

## Related literature   

For the synthesis of anthryl isoxazoles, see: Mosher & Natale (1995 ▶); Zhou *et al.* (1997 ▶); Han & Natale, (2001 ▶); Rider *et al.* (2010 ▶); Mirzaei *et al.* (2012 ▶). For previous studies on anthryl isoxazole crystallography, see: Mosher *et al.* (1996 ▶); Han *et al.* (2002 ▶, 2003 ▶); Li *et al.* (2006 ▶, 2008 ▶). For the anti­tumor activity of aryl isoxazole amides (AIMs), see: Han *et al.* (2009 ▶); Gajewski *et al.* (2009 ▶). For a previous report of a 9′-Br-9′-heterocyclic anthrone crystal structure, see: Riant *et al.* (1994 ▶). 
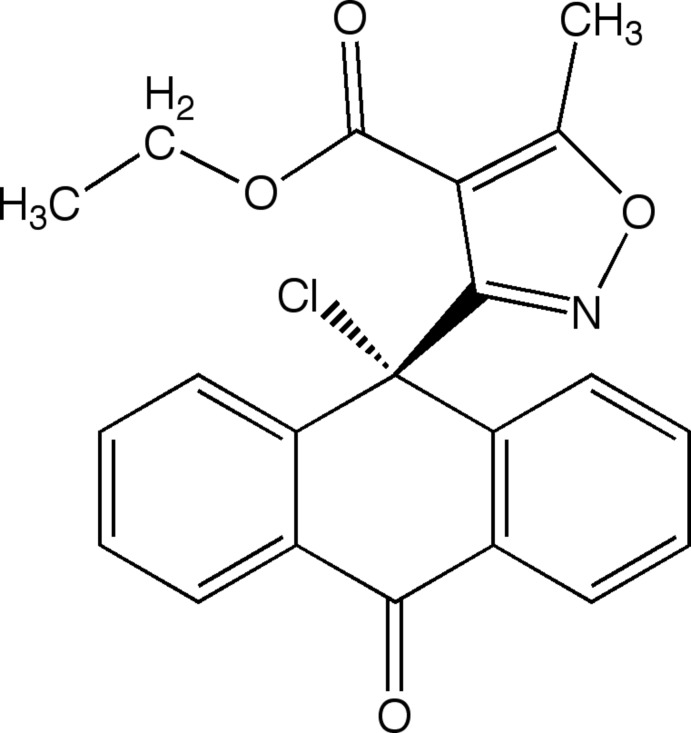


## Experimental   

### 

#### Crystal data  

C_21_H_16_ClNO_4_
*M* *_r_* = 381.80
Triclinic, 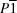

*a* = 10.0121 (3) Å
*b* = 12.6146 (4) Å
*c* = 14.9503 (4) Å
α = 77.9547 (14)°
β = 73.4361 (13)°
γ = 89.1187 (13)°
*V* = 1768.04 (9) Å^3^
*Z* = 4
Mo *K*α radiation
μ = 0.24 mm^−1^
*T* = 100 K
0.47 × 0.37 × 0.23 mm

#### Data collection  

Bruker SMART BREEZE CCD diffractometer
Absorption correction: multi-scan (*SADABS*; Bruker, 2008 ▶) *T*_min_ = 0.89, *T*_max_ = 0.95
48576 measured reflections
8722 independent reflections
8026 reflections with *I* > 2σ(*I*)
*R*_int_ = 0.023

#### Refinement  

*R*[*F* ^2^ > 2σ(*F* ^2^)] = 0.033
*wR*(*F* ^2^) = 0.089
*S* = 1.03
8722 reflections
491 parameters
H-atom parameters constrained
Δρ_max_ = 0.46 e Å^−3^
Δρ_min_ = −0.22 e Å^−3^

### 

Data collection: *APEX2* (Bruker, 2008 ▶); cell refinement: *SAINT* (Bruker, 2008 ▶); data reduction: *SAINT*; program(s) used to solve structure: *SHELXS97* (Sheldrick 2008 ▶); program(s) used to refine structure: *SHELXL2013* (Sheldrick 2008 ▶); molecular graphics: *OLEX2* (Dolomanov *et al.*, 2009 ▶); software used to prepare material for publication: *publCIF* (Westrip, 2010 ▶) and *OLEX2*.

## Supplementary Material

Crystal structure: contains datablock(s) global, I. DOI: 10.1107/S1600536814003080/ff2125sup1.cif

Structure factors: contains datablock(s) I. DOI: 10.1107/S1600536814003080/ff2125Isup2.hkl

Supporting information file. DOI: 10.1107/S1600536814003080/ff2125Isup3.cml

CCDC reference: 986226

Additional supporting information:  crystallographic information; 3D view; checkCIF report

## Figures and Tables

**Table 1:** Hydrogen-bond geometry (Å, °)

*D*—H⋯*A*	*D*—H	H⋯*A*	*D*⋯*A*	*D*—H⋯*A*
C2—H2⋯O1′	0.95	2.55	3.4902 (15)	171
C7—H7⋯N1′^i^	0.95	2.47	3.3294 (15)	151
C1′—H1′⋯O2′^ii^	0.95	2.57	3.4866 (12)	161
C2′—H2′⋯N1^iii^	0.95	2.47	3.3041 (18)	146
C6′—H6′⋯O3′^iv^	0.95	2.49	3.3224 (15)	146
C7′—H7′⋯O1^v^	0.95	2.50	3.4275 (17)	167

Symmetry codes: (i) 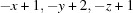; (ii) 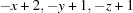; (iii) 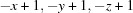; (iv) 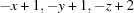; (v) 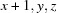.

**Fig. 1.**
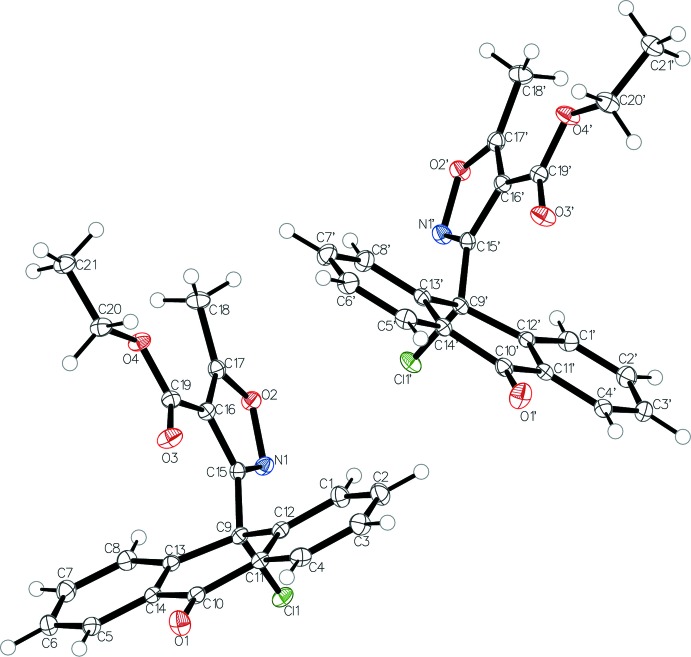
The molecular structure of the title compound, with atom labels and 50% probability displacement ellipsoids for non-H atoms.

## supplementary crystallographic information

### 1. Comment

Isoxazolyl-anthracenyl amides (AIMs) have been found to possess significant antitumor activity (Han *et al.*, 2009; Gajewski *et al.*, 2009), and the title compound was isolated in the course of our continuing structure activity relationship studies. The edge-to-face π-stacking in the unit cell is noteworthy, as the current working hypothesis for the AIMs is that they exert their biological effect via an interaction with G-quadruplex DNA, by an analogous plausible stacking interaction. The title compound exhibited no cytotoxicity up to 25µM in an assay against human glioblastoma SNB-19 cells. However, the structural data in this report will be useful for interpretation of SAR studies with the AIMs that will be reported in due course.

The asymmetric unit of the title compound, C_21_H_16_NO_4_Cl, contains two independent molecules, each adopting a conformation wherein the isoxazole ring is roughly orthogonal to the anthrone ring. The angle between the mean plane of the isoxazole (all atoms) and the mean plane of the anthrone (all atoms) is 91.52 (3)° in one molecule and 90.08 (4)° in the other. The ester is almost co-planar with the isoxazole, with the mean plane angles of 2.48 (15)° and 8.62 (5)°. In both molecules, the distances between isoxazolyl ester carbonyl oxygen and the anthrone carbon of the ketone are about 3.3 Å in distance (O3-C10 and O3'-C10'). The anthrone is virtually planar, and adopts a shallow boat conformation, as evidenced by the sum of the six intra- B-ring torsional angles. The smallest intermolecular distance between the anthrone H2 of one molecule of the title compound is 3.1192 (13) Å from C5' of the second molecule, also the closest centroids of the two anthrone A rings is 5.1162 (7) Å in distance (ring C1-C2-C3-C4-C11-C12 and ring C5'-C6'-C7'-C8'-C13'-C14'), with an angle of 57.98 (5)°, consistent with an edge-to-face π-stacking interaction.

### 2. Experimental

To a suspension of anthracene-9-carbaldehyde (4.0 g, 19.39 mmol; Sigma-Aldrich, 97%) in THF: Ethanol: H_2_O (54 mL: 27 mL: 27 mL) was dissolved sodium acetate (3.5 eq., 5.57 g, 67.89 mmol) and hydroxylamine hydrochloride (2 eq, 2.69 g, 38.71 mmol). The reaction was covered with a septa and let stir at room temperature until TLC showed no starting material remained (ca. 49 hours). The solution was then transferred to a separatory funnel and washed 4 x 200 mL Brine and the combined aqueous layers washed 2 x 50 mL CHCl_3_, dried over sodium sulfate, filtered, and the solvent removed under vacuum to yield anthracene-9-carbaldehyde oxime (99%).^1^H NMR(CDCl_3_) δ 9.22 (s, 1H), 8.51 (s, 1H), 8.42 (d, J=8.66 Hz, 2H), 8.03 (d, J=8.16 Hz, 2H), 7.50-7.59 (m, 4H).

The anthracene-9-carbaldehyde oxime (4.667 g, 21.09 mmol) was taken up in 120 mL of chloroform at room temperature, to which solution was added pyridine (10 mol%, 2.00 mL) and recrystallized NCS (1.1 eq, 3.098 g, 23.20 mmol). The solution brought to 45°C for 3.5 hours then cooled to room temperature. The organic layer was washed with 4 x 200 mL Brine and 2 x 150 mL H_2_O, then the aqueous layer washed 2 x 150 mL CHCl_3_, dried with sodium sulfate, filtered, and the solvent removed under reduced pressure to yield the nitrile oxide. The intermediate was purified only through extractive isolation using brine and CHCl_3_ and taken on to the next reaction as is. To a solution of the nitrile oxide in absolute ethanol (100 mL) was added 1.4 equivalents of ethylacetoacetate (3.8425 g, 29.53 mmol). In a separate round bottom was added 50 mL absolute ethanol and 1.018 g Na(s). Once the sodium dissociation had completed, the warm solution was added to the nitrile oxide and the mixture was allowed to stir at room temperature under argon for 21.5 hours until TLC in 4:1 Hex/EtOAc revealed all nitrile oxide had been consumed. Finally, the ethanol was removed via rotary evaporation and the solid dissolved in CHCl_3_, washed 4 x 200 mL Brine, and the aqueous layer washed 2 x 150 mL CHCl_3_, dried sodium sulfate, and concentrated under reduced pressure. The solid was then chromatographed using 4:1 Hex/EtOAc. Ethyl 3-(anthracen-9-yl)-5-methylisoxazole-4-carboxylate. Yield 93%.^1^H NMR(400 MHz, CDCl_3_) δ 8.59 (s, 1H), 8.06 (d, J=7.91, 2H), 7.66 (d, J=8.16 Hz, 2H), 7.41-7.50 (m, 4H), 3.70 (q, J=7.15 Hz, 2H), 2.93 (s, 3H), 0.33 (t, J=7.15 Hz, 3H). Spectral data are in accord with those reported previously. (Mirzaei, *et al.*, 2012)

The carboxylate (0.300 g, 0.905 mmol) was taken up in 10 mL DMF to which was added a solution over 10 minutes of N-Chlorosuccinimide (NCS) (1.2 eq, 0.1461 g, 1.094 mmol) dissolved in 5 mL DMF. The solution was brought to 43°C and let stir for 4 hours whereupon the solution was poured into 50 mL ice/water which was allowed to stir for 1 hour, in which the 10-Cl carboxylate precipitated out, filtered and washed with 2 x 25 mL water. Yield 96%.^1^H NMR(400 MHz, CDCl_3_) δ 8.59 (d, J=8.91 Hz, 2H), 7.59-7.66 (m, 4H), 7.46-7.50 (m, 2H), 3.72 (q, J=7.15 Hz, 2H), 2.93 (s, 3H), 0.39 (t, J=7.15 Hz, 3H). Spectral data are in accord with those reported previously. (Mirzaei, *et al.*, 2012).

The 10-Cl carboxylate (0.3312 g, 0.905 mmol) was taken up in 5 mL DMF to which was added a solution over 10 minutes of N-Chlorosuccinimide (NCS) (1.2 eq, 0.1451 g, 1.087 mmol) dissolved in 5 mL DMF. The solution was brought to 30°C and let stir for 43 hours whereupon the solution was poured into 50 mL ice/water which was allowed to stir for 1.5 hours, in which the product precipitated out. Product was filtered and washed with water. The solid was dissolved in minimal CH_2_Cl_2_ and placed on a wet silica column prepared with hexanes. The solvent polarity increased using a stepwise elution of 10:1, 6:1, and finally 4:1 until all product collected. Ethyl 3-(9-chloro-10-oxo-9,10-dihydroanthracen-9-yl)-5-methylisoxazole-4-χarboxylate. Single crystals with sufficient quality for X-ray crystallographic analysis were prepared by a slow recrystallization from a chloroform/pentane (3:1) solution. Yield 29%, ^1^H NMR(CDCl_3_) δ 8.38 (dd, J=1.38, 7.65 Hz, 2H), 7.51-7.61 (m, 4H), 7.34 (dd, J=1.13, 7.91 Hz, 2H), 3.56 (q, J=7.15 Hz, 2H), 2.70 (s, 3H), 0.65 (t, J=7.03 Hz, 3H); ^13^C NMR(CDCl_3_) δ 182.58, 178.50,162.69, 159.76, 142.41, 133.92, 129.85, 129.13, 127.84, 127.58, 107.92, 62.51, 60.43, 13.78, 13.47. MS (EI) *m/z* 346(100, M-Cl), 347(28.33, M-Cl)^+^, 348(8.42, M-Cl)^+2^.

### 3. Refinement

All H atoms were placed at geometrically calculated positions and included in the refinement in the riding model approximation, with C—H lengths of 0.93 (aromatic CH), 0.96 (CH3) and 0.97 (CH2) Å. *U*_iso_ of the H atoms was set at 1.5*U*_eq_ of the parent C atom for the methyl group and at 1.2*U*_eq_ for the remaining H atoms.

### Crystal data

**Table:**

C_21_H_16_ClNO_4_	*Z* = 4
*M**_r_* = 381.80	*F*(000) = 792
Triclinic, *P*1	*D*_x_ = 1.434 Mg m^−^^3^
*a* = 10.0121 (3) Å	Mo *K*α radiation, λ = 0.71073 Å
*b* = 12.6146 (4) Å	Cell parameters from 3126 reflections
*c* = 14.9503 (4) Å	µ = 0.24 mm^−^^1^
α = 77.9547 (14)°	*T* = 100 K
β = 73.4361 (13)°	Prism, translucent white
γ = 89.1187 (13)°	0.47 × 0.37 × 0.23 mm
*V* = 1768.04 (9) Å^3^	

### Data collection

**Table:**

Bruker SMART BREEZE CCD diffractometer	8722 independent reflections
Radiation source: 2 kW sealed X-ray tube, 2 kW sealed X-ray tube	8026 reflections with *I* > 2σ(*I*)
Graphite monochromator	*R*_int_ = 0.023
Detector resolution: 8.3333 pixels mm^-1^	θ_max_ = 28.3°, θ_min_ = 1.5°
φ and ω scans	*h* = −12→13
Absorption correction: multi-scan (*SADABS*; Bruker, 2008)	*k* = −16→16
*T*_min_ = 0.89, *T*_max_ = 0.95	*l* = 0→19
48576 measured reflections	

### Refinement

**Table:**

Refinement on *F*^2^	Primary atom site location: structure-invariant direct methods
Least-squares matrix: full	Secondary atom site location: difference Fourier map
*R*[*F*^2^ > 2σ(*F*^2^)] = 0.033	Hydrogen site location: inferred from neighbouring sites
*wR*(*F*^2^) = 0.089	H-atom parameters constrained
*S* = 1.03	*w* = 1/[σ^2^(*F*_o_^2^) + (0.0452*P*)^2^ + 0.9406*P*] where *P* = (*F*_o_^2^ + 2*F*_c_^2^)/3
8722 reflections	(Δ/σ)_max_ = 0.001
491 parameters	Δρ_max_ = 0.46 e Å^−^^3^
0 restraints	Δρ_min_ = −0.22 e Å^−^^3^

### Special details

**Table:**

Geometry. All e.s.d.'s (except the e.s.d. in the dihedral angle between two l.s. planes) are estimated using the full covariance matrix. The cell e.s.d.'s are taken into account individually in the estimation of e.s.d.'s in distances, angles and torsion angles; correlations between e.s.d.'s in cell parameters are only used when they are defined by crystal symmetry. An approximate (isotropic) treatment of cell e.s.d.'s is used for estimating e.s.d.'s involving l.s. planes.
Refinement. Refinement of *F*^2^ against ALL reflections. The weighted *R*-factor *wR* and goodness of fit *S* are based on *F*^2^, conventional *R*-factors *R* are based on *F*, with *F* set to zero for negative *F*^2^. The threshold expression of *F*^2^ > σ(*F*^2^) is used only for calculating *R*-factors(gt) *etc*. and is not relevant to the choice of reflections for refinement. *R*-factors based on *F*^2^ are statistically about twice as large as those based on *F*, and *R*- factors based on ALL data will be even larger.

### Fractional atomic coordinates and isotropic or equivalent isotropic displacement parameters (Å^2^)

**Table:**

	*x*	*y*	*z*	*U*_iso_*/*U*_eq_	
C1	0.14668 (12)	0.69710 (9)	0.77157 (9)	0.0171 (2)	
H1	0.2322	0.6874	0.7267	0.021*	
C2	0.08750 (13)	0.61345 (10)	0.84944 (9)	0.0199 (2)	
H2	0.1321	0.5465	0.8572	0.024*	
C3	−0.03702 (13)	0.62764 (10)	0.91595 (9)	0.0194 (2)	
H3	−0.0774	0.5704	0.9691	0.023*	
C4	−0.10204 (12)	0.72499 (10)	0.90473 (8)	0.0161 (2)	
H4	−0.1866	0.7347	0.9506	0.019*	
C5	−0.11310 (12)	1.10305 (9)	0.72727 (9)	0.0162 (2)	
H5	−0.2003	1.1108	0.7714	0.019*	
C6	−0.05040 (13)	1.19009 (9)	0.65439 (9)	0.0190 (2)	
H6	−0.0957	1.2568	0.6475	0.023*	
C7	0.07903 (13)	1.17969 (10)	0.59119 (9)	0.0192 (2)	
H7	0.1236	1.2401	0.5426	0.023*	
C8	0.14296 (12)	1.08127 (9)	0.59904 (8)	0.0163 (2)	
H8	0.2311	1.0744	0.5556	0.02*	
C9	0.14299 (11)	0.88266 (9)	0.67070 (8)	0.0121 (2)	
C10	−0.11608 (11)	0.91308 (9)	0.81700 (8)	0.0137 (2)	
C11	−0.04392 (11)	0.80947 (9)	0.82605 (8)	0.0128 (2)	
C12	0.08113 (11)	0.79510 (9)	0.75903 (8)	0.0127 (2)	
C13	0.07843 (11)	0.99208 (9)	0.67055 (8)	0.0125 (2)	
C14	−0.04852 (11)	1.00370 (9)	0.73628 (8)	0.0126 (2)	
C15	0.30024 (11)	0.89485 (8)	0.64771 (8)	0.0121 (2)	
C16	0.37774 (11)	0.93407 (9)	0.70223 (8)	0.0130 (2)	
C17	0.51406 (12)	0.93268 (9)	0.64969 (8)	0.0140 (2)	
C18	0.65071 (12)	0.96354 (10)	0.65966 (9)	0.0191 (2)	
H9	0.7208	0.9139	0.6345	0.029*	
H11	0.6424	0.9591	0.7273	0.029*	
H10	0.6791	1.0379	0.6238	0.029*	
C19	0.31997 (12)	0.96906 (9)	0.79302 (8)	0.0139 (2)	
C20	0.36925 (12)	1.05664 (10)	0.90633 (8)	0.0167 (2)	
H12	0.3349	0.9956	0.9619	0.02*	
H13	0.2924	1.1062	0.9031	0.02*	
C21	0.49282 (13)	1.11622 (10)	0.91531 (9)	0.0189 (2)	
H14	0.5705	1.0677	0.9133	0.028*	
H16	0.4671	1.14	0.9761	0.028*	
H15	0.5211	1.1796	0.8624	0.028*	
C1'	0.65302 (12)	0.38133 (10)	0.59466 (8)	0.0169 (2)	
H1'	0.7394	0.4118	0.5514	0.02*	
C2'	0.59357 (13)	0.28905 (10)	0.58200 (9)	0.0195 (2)	
H2'	0.6396	0.2563	0.5304	0.023*	
C3'	0.46644 (13)	0.24425 (10)	0.64482 (9)	0.0193 (2)	
H3'	0.4242	0.1824	0.635	0.023*	
C4'	0.40195 (12)	0.29011 (9)	0.72152 (9)	0.0163 (2)	
H4'	0.3161	0.2587	0.765	0.02*	
C5'	0.40299 (12)	0.56283 (10)	0.91808 (8)	0.0172 (2)	
H5'	0.3222	0.5268	0.9646	0.021*	
C6'	0.46182 (13)	0.65393 (10)	0.93270 (9)	0.0198 (2)	
H6'	0.4215	0.6804	0.989	0.024*	
C7'	0.58021 (13)	0.70657 (10)	0.86465 (9)	0.0201 (2)	
H7'	0.6195	0.7701	0.8738	0.024*	
C8'	0.64117 (12)	0.66661 (10)	0.78341 (9)	0.0180 (2)	
H8'	0.7234	0.7018	0.7381	0.022*	
C9'	0.64736 (11)	0.53627 (8)	0.67675 (8)	0.0119 (2)	
C10'	0.39380 (12)	0.42679 (9)	0.82106 (8)	0.0142 (2)	
C11'	0.46221 (11)	0.38264 (9)	0.73568 (8)	0.0128 (2)	
C12'	0.58679 (11)	0.42963 (9)	0.67049 (8)	0.0125 (2)	
C13'	0.58229 (11)	0.57506 (9)	0.76802 (8)	0.0128 (2)	
C14'	0.46152 (11)	0.52325 (9)	0.83524 (8)	0.0132 (2)	
C15'	0.80463 (11)	0.53349 (9)	0.65468 (8)	0.0124 (2)	
C16'	0.88436 (11)	0.46614 (9)	0.70831 (8)	0.0129 (2)	
C17'	1.01975 (12)	0.49193 (9)	0.65518 (8)	0.0147 (2)	
C18'	1.15761 (12)	0.45330 (10)	0.66295 (9)	0.0201 (2)	
H11'	1.1876	0.3992	0.6238	0.03*	
H10'	1.1501	0.4205	0.7298	0.03*	
H9'	1.2261	0.5147	0.6405	0.03*	
C19'	0.82856 (12)	0.38530 (9)	0.79801 (8)	0.0136 (2)	
C20'	0.88427 (13)	0.24866 (10)	0.91601 (9)	0.0195 (2)	
H12'	0.804	0.2051	0.9142	0.023*	
H13'	0.8555	0.281	0.9731	0.023*	
C21'	1.00659 (13)	0.17823 (10)	0.91951 (9)	0.0215 (2)	
H14'	1.0301	0.143	0.8648	0.032*	
H16'	0.9821	0.1227	0.9789	0.032*	
H15'	1.087	0.2232	0.9171	0.032*	
Cl1	0.10081 (3)	0.83526 (2)	0.573222 (19)	0.01709 (7)	
Cl1'	0.60533 (3)	0.63770 (2)	0.58213 (2)	0.01801 (7)	
N1	0.38204 (10)	0.87205 (8)	0.56959 (7)	0.01485 (19)	
N1'	0.88548 (10)	0.59449 (8)	0.57680 (7)	0.01584 (19)	
O1	−0.22728 (9)	0.92378 (7)	0.87479 (6)	0.02125 (18)	
O2	0.51896 (8)	0.89548 (7)	0.57090 (6)	0.01550 (16)	
O3	0.19812 (9)	0.95794 (8)	0.83804 (6)	0.02023 (18)	
O4	0.41820 (8)	1.01639 (7)	0.81836 (6)	0.01672 (17)	
O1'	0.28444 (9)	0.38481 (7)	0.87812 (6)	0.02227 (19)	
O2'	1.02297 (8)	0.56851 (7)	0.57716 (6)	0.01688 (17)	
O3'	0.70532 (9)	0.36690 (7)	0.83742 (6)	0.01982 (18)	
O4'	0.92966 (9)	0.33385 (7)	0.82938 (6)	0.01871 (18)	

### Atomic displacement parameters (Å^2^)

**Table:**

	*U*^11^	*U*^22^	*U*^33^	*U*^12^	*U*^13^	*U*^23^
C1	0.0149 (5)	0.0158 (5)	0.0182 (6)	0.0020 (4)	−0.0012 (4)	−0.0031 (4)
C2	0.0197 (6)	0.0150 (5)	0.0230 (6)	0.0021 (4)	−0.0053 (5)	−0.0008 (4)
C3	0.0184 (6)	0.0173 (5)	0.0185 (6)	−0.0025 (4)	−0.0041 (4)	0.0034 (4)
C4	0.0131 (5)	0.0190 (5)	0.0143 (5)	−0.0016 (4)	−0.0023 (4)	−0.0014 (4)
C5	0.0151 (5)	0.0160 (5)	0.0194 (6)	0.0020 (4)	−0.0059 (4)	−0.0067 (4)
C6	0.0232 (6)	0.0138 (5)	0.0226 (6)	0.0030 (4)	−0.0105 (5)	−0.0043 (4)
C7	0.0240 (6)	0.0150 (5)	0.0180 (6)	−0.0022 (4)	−0.0076 (5)	0.0003 (4)
C8	0.0163 (5)	0.0167 (5)	0.0143 (5)	−0.0013 (4)	−0.0032 (4)	−0.0012 (4)
C9	0.0127 (5)	0.0134 (5)	0.0105 (5)	−0.0005 (4)	−0.0027 (4)	−0.0045 (4)
C10	0.0123 (5)	0.0147 (5)	0.0146 (5)	−0.0007 (4)	−0.0036 (4)	−0.0049 (4)
C11	0.0121 (5)	0.0136 (5)	0.0129 (5)	−0.0011 (4)	−0.0038 (4)	−0.0031 (4)
C12	0.0124 (5)	0.0126 (5)	0.0131 (5)	−0.0017 (4)	−0.0035 (4)	−0.0025 (4)
C13	0.0136 (5)	0.0127 (5)	0.0125 (5)	0.0002 (4)	−0.0055 (4)	−0.0031 (4)
C14	0.0123 (5)	0.0128 (5)	0.0139 (5)	−0.0004 (4)	−0.0050 (4)	−0.0039 (4)
C15	0.0128 (5)	0.0108 (5)	0.0117 (5)	0.0003 (4)	−0.0020 (4)	−0.0023 (4)
C16	0.0126 (5)	0.0131 (5)	0.0125 (5)	−0.0001 (4)	−0.0030 (4)	−0.0023 (4)
C17	0.0145 (5)	0.0130 (5)	0.0136 (5)	0.0008 (4)	−0.0031 (4)	−0.0020 (4)
C18	0.0118 (5)	0.0250 (6)	0.0201 (6)	−0.0002 (4)	−0.0039 (4)	−0.0048 (5)
C19	0.0145 (5)	0.0152 (5)	0.0119 (5)	0.0002 (4)	−0.0043 (4)	−0.0021 (4)
C20	0.0157 (5)	0.0235 (6)	0.0131 (5)	0.0019 (4)	−0.0043 (4)	−0.0085 (4)
C21	0.0190 (6)	0.0225 (6)	0.0182 (6)	0.0002 (4)	−0.0076 (4)	−0.0077 (4)
C1'	0.0156 (5)	0.0202 (5)	0.0150 (5)	0.0025 (4)	−0.0030 (4)	−0.0057 (4)
C2'	0.0224 (6)	0.0210 (6)	0.0184 (6)	0.0058 (5)	−0.0072 (5)	−0.0100 (5)
C3'	0.0236 (6)	0.0147 (5)	0.0233 (6)	0.0019 (4)	−0.0112 (5)	−0.0063 (4)
C4'	0.0153 (5)	0.0134 (5)	0.0203 (6)	0.0008 (4)	−0.0065 (4)	−0.0018 (4)
C5'	0.0138 (5)	0.0214 (6)	0.0146 (5)	0.0024 (4)	−0.0005 (4)	−0.0050 (4)
C6'	0.0180 (6)	0.0247 (6)	0.0184 (6)	0.0041 (5)	−0.0032 (4)	−0.0116 (5)
C7'	0.0180 (6)	0.0202 (6)	0.0246 (6)	0.0006 (4)	−0.0052 (5)	−0.0112 (5)
C8'	0.0149 (5)	0.0177 (5)	0.0202 (6)	−0.0013 (4)	−0.0016 (4)	−0.0061 (4)
C9'	0.0118 (5)	0.0114 (5)	0.0108 (5)	0.0017 (4)	−0.0023 (4)	−0.0004 (4)
C10'	0.0127 (5)	0.0136 (5)	0.0148 (5)	0.0017 (4)	−0.0026 (4)	−0.0019 (4)
C11'	0.0123 (5)	0.0126 (5)	0.0138 (5)	0.0027 (4)	−0.0046 (4)	−0.0021 (4)
C12'	0.0124 (5)	0.0125 (5)	0.0131 (5)	0.0023 (4)	−0.0049 (4)	−0.0026 (4)
C13'	0.0119 (5)	0.0130 (5)	0.0135 (5)	0.0033 (4)	−0.0032 (4)	−0.0037 (4)
C14'	0.0123 (5)	0.0132 (5)	0.0137 (5)	0.0025 (4)	−0.0029 (4)	−0.0031 (4)
C15'	0.0127 (5)	0.0111 (5)	0.0127 (5)	0.0011 (4)	−0.0021 (4)	−0.0034 (4)
C16'	0.0120 (5)	0.0134 (5)	0.0132 (5)	0.0014 (4)	−0.0030 (4)	−0.0034 (4)
C17'	0.0146 (5)	0.0140 (5)	0.0149 (5)	0.0006 (4)	−0.0028 (4)	−0.0042 (4)
C18'	0.0119 (5)	0.0253 (6)	0.0222 (6)	0.0023 (4)	−0.0030 (4)	−0.0057 (5)
C19'	0.0141 (5)	0.0152 (5)	0.0122 (5)	0.0023 (4)	−0.0039 (4)	−0.0041 (4)
C20'	0.0178 (6)	0.0216 (6)	0.0152 (5)	0.0019 (4)	−0.0037 (4)	0.0032 (4)
C21'	0.0222 (6)	0.0218 (6)	0.0215 (6)	0.0057 (5)	−0.0098 (5)	−0.0019 (5)
Cl1	0.01746 (13)	0.02050 (13)	0.01522 (13)	−0.00197 (10)	−0.00467 (10)	−0.00783 (10)
Cl1'	0.01790 (13)	0.01718 (13)	0.01622 (13)	0.00446 (10)	−0.00449 (10)	0.00158 (10)
N1	0.0111 (4)	0.0161 (4)	0.0167 (5)	−0.0013 (3)	−0.0018 (4)	−0.0051 (4)
N1'	0.0107 (4)	0.0161 (4)	0.0174 (5)	0.0014 (3)	−0.0004 (4)	−0.0016 (4)
O1	0.0162 (4)	0.0198 (4)	0.0222 (4)	0.0021 (3)	0.0029 (3)	−0.0042 (3)
O2	0.0117 (4)	0.0176 (4)	0.0167 (4)	0.0004 (3)	−0.0016 (3)	−0.0061 (3)
O3	0.0139 (4)	0.0301 (5)	0.0175 (4)	−0.0022 (3)	−0.0015 (3)	−0.0106 (4)
O4	0.0138 (4)	0.0241 (4)	0.0140 (4)	−0.0007 (3)	−0.0032 (3)	−0.0087 (3)
O1'	0.0176 (4)	0.0215 (4)	0.0219 (4)	−0.0054 (3)	0.0042 (3)	−0.0053 (3)
O2'	0.0110 (4)	0.0172 (4)	0.0184 (4)	0.0004 (3)	−0.0001 (3)	−0.0009 (3)
O3'	0.0132 (4)	0.0248 (4)	0.0165 (4)	0.0020 (3)	−0.0016 (3)	0.0025 (3)
O4'	0.0134 (4)	0.0216 (4)	0.0171 (4)	0.0019 (3)	−0.0040 (3)	0.0040 (3)

### Geometric parameters (Å, º)

**Table:**

C1—H1	0.95	C1'—C2'	1.3866 (17)
C1—C2	1.3903 (16)	C1'—C12'	1.3940 (15)
C1—C12	1.3938 (15)	C2'—H2'	0.95
C2—H2	0.95	C2'—C3'	1.3931 (18)
C2—C3	1.3913 (17)	C3'—H3'	0.95
C3—H3	0.95	C3'—C4'	1.3820 (17)
C3—C4	1.3826 (17)	C4'—H4'	0.95
C4—H4	0.95	C4'—C11'	1.4031 (15)
C4—C11	1.4012 (15)	C5'—H5'	0.95
C5—H5	0.95	C5'—C6'	1.3835 (17)
C5—C6	1.3852 (17)	C5'—C14'	1.4007 (16)
C5—C14	1.4004 (15)	C6'—H6'	0.95
C6—H6	0.95	C6'—C7'	1.3913 (17)
C6—C7	1.3920 (18)	C7'—H7'	0.95
C7—H7	0.95	C7'—C8'	1.3875 (17)
C7—C8	1.3865 (17)	C8'—H8'	0.95
C8—H8	0.95	C8'—C13'	1.3941 (16)
C8—C13	1.3975 (15)	C9'—C12'	1.5153 (15)
C9—C12	1.5144 (15)	C9'—C13'	1.5125 (15)
C9—C13	1.5150 (15)	C9'—C15'	1.5154 (15)
C9—C15	1.5157 (15)	C9'—Cl1'	1.8392 (11)
C9—Cl1	1.8390 (11)	C10'—C11'	1.4816 (15)
C10—C11	1.4837 (15)	C10'—C14'	1.4834 (15)
C10—C14	1.4843 (15)	C10'—O1'	1.2263 (14)
C10—O1	1.2247 (14)	C11'—C12'	1.3948 (15)
C11—C12	1.3993 (15)	C13'—C14'	1.3986 (15)
C13—C14	1.3947 (15)	C15'—C16'	1.4358 (15)
C15—C16	1.4324 (15)	C15'—N1'	1.3080 (14)
C15—N1	1.3058 (14)	C16'—C17'	1.3660 (15)
C16—C17	1.3689 (15)	C16'—C19'	1.4732 (15)
C16—C19	1.4718 (15)	C17'—C18'	1.4844 (16)
C17—C18	1.4840 (16)	C17'—O2'	1.3431 (14)
C17—O2	1.3443 (14)	C18'—H11'	0.98
C18—H9	0.98	C18'—H10'	0.98
C18—H11	0.98	C18'—H9'	0.98
C18—H10	0.98	C19'—O3'	1.2086 (14)
C19—O3	1.2078 (14)	C19'—O4'	1.3376 (13)
C19—O4	1.3387 (13)	C20'—H12'	0.99
C20—H12	0.99	C20'—H13'	0.99
C20—H13	0.99	C20'—C21'	1.5085 (17)
C20—C21	1.5096 (16)	C20'—O4'	1.4620 (14)
C20—O4	1.4598 (13)	C21'—H14'	0.98
C21—H14	0.98	C21'—H16'	0.98
C21—H16	0.98	C21'—H15'	0.98
C21—H15	0.98	N1—O2	1.4132 (12)
C1'—H1'	0.95	N1'—O2'	1.4114 (12)
			
C2—C1—H1	119.8	C1'—C2'—H2'	119.9
C2—C1—C12	120.35 (11)	C1'—C2'—C3'	120.12 (11)
C12—C1—H1	119.8	C3'—C2'—H2'	119.9
C1—C2—H2	120.0	C2'—C3'—H3'	120.1
C1—C2—C3	120.05 (11)	C4'—C3'—C2'	119.75 (11)
C3—C2—H2	120.0	C4'—C3'—H3'	120.1
C2—C3—H3	120.0	C3'—C4'—H4'	119.7
C4—C3—C2	120.04 (11)	C3'—C4'—C11'	120.54 (11)
C4—C3—H3	120.0	C11'—C4'—H4'	119.7
C3—C4—H4	119.8	C6'—C5'—H5'	119.7
C3—C4—C11	120.36 (11)	C6'—C5'—C14'	120.54 (11)
C11—C4—H4	119.8	C14'—C5'—H5'	119.7
C6—C5—H5	119.8	C5'—C6'—H6'	120.1
C6—C5—C14	120.31 (11)	C5'—C6'—C7'	119.75 (11)
C14—C5—H5	119.8	C7'—C6'—H6'	120.1
C5—C6—H6	120.0	C6'—C7'—H7'	119.9
C5—C6—C7	119.91 (11)	C8'—C7'—C6'	120.25 (11)
C7—C6—H6	120.0	C8'—C7'—H7'	119.9
C6—C7—H7	120.0	C7'—C8'—H8'	119.8
C8—C7—C6	120.10 (11)	C7'—C8'—C13'	120.32 (11)
C8—C7—H7	120.0	C13'—C8'—H8'	119.8
C7—C8—H8	119.8	C12'—C9'—C15'	110.41 (9)
C7—C8—C13	120.35 (11)	C12'—C9'—Cl1'	104.74 (7)
C13—C8—H8	119.8	C13'—C9'—C12'	115.68 (9)
C12—C9—C13	115.60 (9)	C13'—C9'—C15'	111.79 (9)
C12—C9—C15	111.79 (9)	C13'—C9'—Cl1'	105.16 (7)
C12—C9—Cl1	104.99 (7)	C15'—C9'—Cl1'	108.46 (7)
C13—C9—C15	110.64 (9)	C11'—C10'—C14'	117.98 (10)
C13—C9—Cl1	104.61 (7)	O1'—C10'—C11'	120.91 (10)
C15—C9—Cl1	108.58 (7)	O1'—C10'—C14'	121.11 (10)
C11—C10—C14	117.91 (10)	C4'—C11'—C10'	119.18 (10)
O1—C10—C11	121.04 (10)	C12'—C11'—C4'	119.46 (10)
O1—C10—C14	121.05 (10)	C12'—C11'—C10'	121.34 (10)
C4—C11—C10	118.76 (10)	C1'—C12'—C9'	119.00 (10)
C12—C11—C4	119.60 (10)	C1'—C12'—C11'	119.64 (10)
C12—C11—C10	121.63 (10)	C11'—C12'—C9'	121.26 (10)
C1—C12—C9	119.43 (10)	C8'—C13'—C9'	119.14 (10)
C1—C12—C11	119.59 (10)	C8'—C13'—C14'	119.61 (10)
C11—C12—C9	120.91 (10)	C14'—C13'—C9'	121.21 (10)
C8—C13—C9	119.07 (10)	C5'—C14'—C10'	119.01 (10)
C14—C13—C8	119.54 (10)	C13'—C14'—C5'	119.49 (10)
C14—C13—C9	121.25 (10)	C13'—C14'—C10'	121.50 (10)
C5—C14—C10	119.02 (10)	C16'—C15'—C9'	127.97 (10)
C13—C14—C5	119.70 (10)	N1'—C15'—C9'	120.52 (10)
C13—C14—C10	121.27 (10)	N1'—C15'—C16'	111.46 (10)
C16—C15—C9	127.17 (10)	C15'—C16'—C19'	126.54 (10)
N1—C15—C9	121.03 (10)	C17'—C16'—C15'	104.15 (10)
N1—C15—C16	111.76 (10)	C17'—C16'—C19'	129.26 (10)
C15—C16—C19	126.63 (10)	C16'—C17'—C18'	135.17 (11)
C17—C16—C15	104.16 (10)	O2'—C17'—C16'	109.33 (10)
C17—C16—C19	129.20 (10)	O2'—C17'—C18'	115.47 (10)
C16—C17—C18	135.08 (11)	C17'—C18'—H11'	109.5
O2—C17—C16	109.11 (10)	C17'—C18'—H10'	109.5
O2—C17—C18	115.80 (10)	C17'—C18'—H9'	109.5
C17—C18—H9	109.5	H11'—C18'—H10'	109.5
C17—C18—H11	109.5	H11'—C18'—H9'	109.5
C17—C18—H10	109.5	H10'—C18'—H9'	109.5
H9—C18—H11	109.5	O3'—C19'—C16'	123.46 (10)
H9—C18—H10	109.5	O3'—C19'—O4'	124.30 (10)
H11—C18—H10	109.5	O4'—C19'—C16'	112.24 (9)
O3—C19—C16	123.82 (10)	H12'—C20'—H13'	108.6
O3—C19—O4	124.34 (10)	C21'—C20'—H12'	110.3
O4—C19—C16	111.82 (9)	C21'—C20'—H13'	110.3
H12—C20—H13	108.6	O4'—C20'—H12'	110.3
C21—C20—H12	110.5	O4'—C20'—H13'	110.3
C21—C20—H13	110.5	O4'—C20'—C21'	107.02 (10)
O4—C20—H12	110.5	C20'—C21'—H14'	109.5
O4—C20—H13	110.5	C20'—C21'—H16'	109.5
O4—C20—C21	106.38 (9)	C20'—C21'—H15'	109.5
C20—C21—H14	109.5	H14'—C21'—H16'	109.5
C20—C21—H16	109.5	H14'—C21'—H15'	109.5
C20—C21—H15	109.5	H16'—C21'—H15'	109.5
H14—C21—H16	109.5	C15—N1—O2	105.32 (9)
H14—C21—H15	109.5	C15'—N1'—O2'	105.48 (9)
H16—C21—H15	109.5	C17—O2—N1	109.65 (8)
C2'—C1'—H1'	119.8	C19—O4—C20	115.53 (9)
C2'—C1'—C12'	120.42 (11)	C17'—O2'—N1'	109.57 (8)
C12'—C1'—H1'	119.8	C19'—O4'—C20'	116.25 (9)
			
C1—C2—C3—C4	−0.03 (19)	C7'—C8'—C13'—C14'	−0.29 (17)
C2—C1—C12—C9	−176.44 (11)	C8'—C13'—C14'—C5'	−1.28 (16)
C2—C1—C12—C11	0.85 (17)	C8'—C13'—C14'—C10'	178.91 (10)
C2—C3—C4—C11	0.61 (18)	C9'—C13'—C14'—C5'	−178.84 (10)
C3—C4—C11—C10	−179.42 (11)	C9'—C13'—C14'—C10'	1.35 (16)
C3—C4—C11—C12	−0.45 (17)	C9'—C15'—C16'—C17'	177.03 (11)
C4—C11—C12—C1	−0.28 (16)	C9'—C15'—C16'—C19'	−0.75 (18)
C4—C11—C12—C9	176.97 (10)	C9'—C15'—N1'—O2'	−177.67 (9)
C5—C6—C7—C8	2.26 (18)	C10'—C11'—C12'—C1'	175.55 (10)
C6—C5—C14—C10	178.02 (10)	C10'—C11'—C12'—C9'	−8.31 (16)
C6—C5—C14—C13	−1.02 (16)	C11'—C10'—C14'—C5'	−176.52 (10)
C6—C7—C8—C13	−0.29 (18)	C11'—C10'—C14'—C13'	3.30 (16)
C7—C8—C13—C9	173.37 (10)	C12'—C1'—C2'—C3'	0.40 (18)
C7—C8—C13—C14	−2.33 (17)	C12'—C9'—C13'—C8'	173.61 (10)
C8—C13—C14—C5	2.97 (16)	C12'—C9'—C13'—C14'	−8.82 (15)
C8—C13—C14—C10	−176.04 (10)	C12'—C9'—C15'—C16'	−65.23 (14)
C9—C13—C14—C5	−172.63 (10)	C12'—C9'—C15'—N1'	111.96 (11)
C9—C13—C14—C10	8.35 (15)	C13'—C9'—C12'—C1'	−171.48 (10)
C9—C15—C16—C17	−177.95 (10)	C13'—C9'—C12'—C11'	12.35 (14)
C9—C15—C16—C19	1.07 (18)	C13'—C9'—C15'—C16'	65.06 (14)
C9—C15—N1—O2	178.44 (9)	C13'—C9'—C15'—N1'	−117.75 (11)
C10—C11—C12—C1	178.66 (10)	C14'—C5'—C6'—C7'	−0.10 (18)
C10—C11—C12—C9	−4.09 (16)	C14'—C10'—C11'—C4'	178.70 (10)
C11—C10—C14—C5	−178.71 (10)	C14'—C10'—C11'—C12'	0.25 (15)
C11—C10—C14—C13	0.31 (15)	C15'—C9'—C12'—C1'	−43.29 (13)
C12—C1—C2—C3	−0.70 (19)	C15'—C9'—C12'—C11'	140.54 (10)
C12—C9—C13—C8	170.17 (10)	C15'—C9'—C13'—C8'	46.10 (13)
C12—C9—C13—C14	−14.20 (14)	C15'—C9'—C13'—C14'	−136.33 (10)
C12—C9—C15—C16	−67.49 (14)	C15'—C16'—C17'—C18'	−177.22 (13)
C12—C9—C15—N1	114.81 (11)	C15'—C16'—C17'—O2'	0.65 (12)
C13—C9—C12—C1	−170.74 (10)	C15'—C16'—C19'—O3'	−0.30 (18)
C13—C9—C12—C11	12.01 (14)	C15'—C16'—C19'—O4'	178.59 (10)
C13—C9—C15—C16	62.90 (14)	C15'—N1'—O2'—C17'	0.47 (12)
C13—C9—C15—N1	−114.80 (11)	C16'—C15'—N1'—O2'	−0.06 (12)
C14—C5—C6—C7	−1.60 (17)	C16'—C17'—O2'—N1'	−0.72 (12)
C14—C10—C11—C4	176.47 (10)	C16'—C19'—O4'—C20'	−177.48 (9)
C14—C10—C11—C12	−2.47 (15)	C17'—C16'—C19'—O3'	−177.53 (12)
C15—C9—C12—C1	−42.96 (13)	C17'—C16'—C19'—O4'	1.37 (17)
C15—C9—C12—C11	139.79 (10)	C18'—C17'—O2'—N1'	177.61 (9)
C15—C9—C13—C8	41.83 (13)	C19'—C16'—C17'—C18'	0.5 (2)
C15—C9—C13—C14	−142.55 (10)	C19'—C16'—C17'—O2'	178.35 (10)
C15—C16—C17—C18	178.51 (12)	C21'—C20'—O4'—C19'	161.23 (10)
C15—C16—C17—O2	−0.33 (12)	Cl1—C9—C12—C1	74.57 (11)
C15—C16—C19—O3	8.13 (18)	Cl1—C9—C12—C11	−102.68 (10)
C15—C16—C19—O4	−170.64 (10)	Cl1—C9—C13—C8	−74.92 (11)
C15—N1—O2—C17	−0.62 (11)	Cl1—C9—C13—C14	100.71 (10)
C16—C15—N1—O2	0.41 (12)	Cl1—C9—C15—C16	177.16 (9)
C16—C17—O2—N1	0.60 (12)	Cl1—C9—C15—N1	−0.54 (13)
C16—C19—O4—C20	178.23 (9)	Cl1'—C9'—C12'—C1'	73.28 (11)
C17—C16—C19—O3	−173.10 (12)	Cl1'—C9'—C12'—C11'	−102.89 (10)
C17—C16—C19—O4	8.13 (16)	Cl1'—C9'—C13'—C8'	−71.39 (11)
C18—C17—O2—N1	−178.50 (9)	Cl1'—C9'—C13'—C14'	106.18 (10)
C19—C16—C17—C18	−0.5 (2)	Cl1'—C9'—C15'—C16'	−179.45 (9)
C19—C16—C17—O2	−179.31 (10)	Cl1'—C9'—C15'—N1'	−2.27 (13)
C21—C20—O4—C19	−174.29 (10)	N1—C15—C16—C17	−0.07 (13)
C1'—C2'—C3'—C4'	−2.02 (18)	N1—C15—C16—C19	178.95 (10)
C2'—C1'—C12'—C9'	−174.16 (10)	N1'—C15'—C16'—C17'	−0.36 (13)
C2'—C1'—C12'—C11'	2.07 (17)	N1'—C15'—C16'—C19'	−178.15 (10)
C2'—C3'—C4'—C11'	1.17 (17)	O1—C10—C11—C4	−2.90 (16)
C3'—C4'—C11'—C10'	−177.19 (10)	O1—C10—C11—C12	178.15 (11)
C3'—C4'—C11'—C12'	1.29 (17)	O1—C10—C14—C5	0.67 (16)
C4'—C11'—C12'—C1'	−2.90 (16)	O1—C10—C14—C13	179.69 (11)
C4'—C11'—C12'—C9'	173.24 (10)	O3—C19—O4—C20	−0.53 (16)
C5'—C6'—C7'—C8'	−1.49 (19)	O1'—C10'—C11'—C4'	−1.38 (16)
C6'—C5'—C14'—C10'	−178.70 (11)	O1'—C10'—C11'—C12'	−179.83 (11)
C6'—C5'—C14'—C13'	1.49 (17)	O1'—C10'—C14'—C5'	3.56 (17)
C6'—C7'—C8'—C13'	1.69 (19)	O1'—C10'—C14'—C13'	−176.62 (11)
C7'—C8'—C13'—C9'	177.32 (11)	O3'—C19'—O4'—C20'	1.40 (17)

### Hydrogen-bond geometry (Å, º)

**Table:**

*D*—H···*A*	*D*—H	H···*A*	*D*···*A*	*D*—H···*A*
C2—H2···O1′	0.95	2.55	3.4902 (15)	171
C7—H7···N1′^i^	0.95	2.47	3.3294 (15)	151
C1′—H1′···O2′^ii^	0.95	2.57	3.4866 (12)	161
C2′—H2′···N1^iii^	0.95	2.47	3.3041 (18)	146
C6′—H6′···O3′^iv^	0.95	2.49	3.3224 (15)	146
C7′—H7′···O1^v^	0.95	2.50	3.4275 (17)	167

Symmetry codes: (i) −*x*+1, −*y*+2, −*z*+1; (ii) −*x*+2, −*y*+1, −*z*+1; (iii) −*x*+1, −*y*+1, −*z*+1; (iv) −*x*+1, −*y*+1, −*z*+2; (v) *x*+1, *y*, *z*.
